# Editorial: Biocultural perspectives on oral health disparities

**DOI:** 10.3389/froh.2025.1770466

**Published:** 2026-01-12

**Authors:** Praveen Hoogar, Vinod Bhat, MR Gangadhar

**Affiliations:** 1 School of Social Sciences, The Apollo University, Chittoor, Andhra Pradesh, India; 2 The Apollo University, Chittoor, Andhra Pradesh, India; 3 Chamarajanagara University, Chamarajanagara, Karnataka, India

**Keywords:** biocultural approach, cultural beliefs, health inequities and inclusion, interdisciplinary research (IDR), medical anthropology, oral health disparities, social determinants of health

Despite significant advancements in modern dentistry and public health, oral health disparities remain a persistent global challenge. For too long, the mouth has been treated in isolation from the body, and the patient in isolation from their environment. The failure of purely biomedical approaches to close the gap in oral health outcomes particularly among marginalized and vulnerable communities' points to the need for a more holistic framework. This Research Topic, *Biocultural Perspectives on Oral Health Disparities*, was curated to address this very gap. By adopting a “biocultural” lens, the articles in this Research Topic explore how biological determinants of disease are inextricably tangled with cultural beliefs, social behaviors, and economic structures.

The eight articles contributed to this topic span diverse geographies and populations, yet they share a common thread: the recognition that oral health is a socially produced phenomenon as much as a biological one.

## Bridging theory and practice

To understand disparities, we must first expand our methodological toolkit. In the review (Howard et al.), the authors argue compellingly for the inclusion of social science perspectives in dental research. They suggest that to truly address oral health inequities, researchers must move beyond clinical metrics to understand the lived experiences and social contexts of patients. This theoretical stance is complemented by the review (Maitra et al.). This paper bridges the gap between the micro-level (vitamin D metabolism) and the macro-level (cultural “habitus”), illustrating how deep-seated cultural dispositions can have measurable biological consequences.

## Cultural beliefs and risk behaviors

A core tenet of the biocultural approach is investigating how local beliefs influence health behaviors. Two studies in this collection highlight this dynamic in starkly different contexts. In Nigeria, the study (Foláyan et al.) explores the intersection of traditional beliefs and modern diet. The authors examine how the cultural concept of “Jẹ`díjẹ`dí” interacts with sugar consumption to shape caries risk in children, underscoring the need for culturally sensitive health education that navigates local belief systems. Meanwhile, in India, the study (Siddiqui et al.) provides a grim look at how culturally entrenched habits drive disease. The high prevalence of smokeless tobacco use in this region serves as a reminder that cultural practices can act as powerful vectors for biological harm, requiring interventions that are both culturally grounded and clinically robust.

## Structural barriers: geography and economics

Disparities are often etched into the landscape itself. Several articles in this topic demonstrate how physical and economic barriers dictate oral health outcomes. The study (Hoa et al.) reveals how geographical isolation and ethnicity compound to create high caries prevalence among children who lack access to standard dental care. Similarly, the broad analysis in (dos Santos Soares et al.) confirms that socioeconomic status remains a definitive predictor of oral function. These findings reinforce the reality that biological resilience is often overwhelmed by structural inequality.

## The lived experience of vulnerable populations

Finally, this collection shines a light on populations often left in the shadows of public health policy. The qualitative study (Velpula et al.) offers a voice to the elderly. By documenting their narratives, the authors reveal how aging is not just a biological process of decay but a social experience of navigating a healthcare system that may not prioritize their needs. In a similar vein, the research (Alshatrat et al.) identifies a critical gap in communication. The study finds significant disparities in health knowledge among the hearing-impaired, suggesting that public health messaging is failing to reach those with sensory disabilities.

## From evidence to action: implications of a biocultural approach

Taken together, the contributions to this Research Topic move beyond documenting oral health disparities to interrogating how and why they are produced. The collective evidence presented here underscores the limitations of narrowly biomedical and behaviorist models and highlights the necessity of adopting a biocultural approach that situates oral health within lived social, cultural, and structural realities.

From a policy perspective, these findings suggest that oral health must be more explicitly embedded within broader social and public health agendas. Policies addressing poverty, education, disability inclusion, aging, and geographic access to care should be recognized as integral to oral health equity. Culturally responsive health communication and community engagement emerge as essential components of effective oral health promotion, rather than supplementary strategies.

For clinical practice, the studies in this collection emphasize the importance of reflexivity and cultural competence in patient care. Understanding patients' explanatory models of illness, habitual practices, and structural constraints can strengthen trust, improve adherence, and reduce miscommunication. This is particularly relevant when working with older adults, ethnic minorities, and individuals with sensory disabilities, whose needs are often insufficiently addressed within standard dental care pathways.

Finally, this Research Topic highlights clear directions for future research. Interdisciplinary collaboration between dental sciences, social sciences, public health, and health policy is essential for advancing a more comprehensive understanding of oral health disparities. Methodologically pluralistic approaches that integrate biological markers with qualitative insights can help move the field beyond documenting inequities toward informing interventions that are context-sensitive, equitable, and socially grounded. By foregrounding these intersections, the studies in this collection collectively argue for a reorientation of oral health research and practice toward integrative, sustainable solutions.

## Conclusion

Collectively, these eight articles demonstrate that there is no single “magic bullet” for eliminating oral health disparities. A child in the mountains of Vietnam, an elderly patient in the UK, and a hearing-impaired individual in Jordan face vastly different challenges. However, the solution in all cases requires a biocultural approach—one that respects the biological reality of disease while aggressively addressing the cultural, social, and economic conditions that allow it to thrive.

As editors, we hope this Research Topic serves as a catalyst for future interdisciplinary work. We extend our gratitude to the authors for their diverse contributions and to the reviewers for ensuring the scientific rigor of this collection.

